# Alterations of fractional anisotropy and white matter integrity in irritable bowel syndrome: a systematic review and meta-analysis of diffusion tensor imaging studies

**DOI:** 10.3389/fnins.2024.1426218

**Published:** 2024-12-02

**Authors:** Mahsa Asadi Anar, Aydin Hassanpour Adeh, Samira Peiravi, Alireza Imani Porshokouh, Seyedeh Sara Rezazadeh Shojaee, Farnaz Najafi, Yasamin Pishkari, Arash Rahimi, Shaghayegh Karami

**Affiliations:** ^1^Student Research Committee, School of Medicine, Shahid Beheshti University of Medical Sciences, Tehran, Iran; ^2^Universal Scientific Education and Research Network, Tehran, Iran; ^3^School of Medicine, Islamic Azad University, Tabriz, Iran; ^4^Department of Emergency Medicine, Mashhad University of Medical Sciences, Mashhad, Iran; ^5^School of Medicine, Iran University of Medical Sciences, Tehran, Iran; ^6^Department of Nursing, Faculty of Nursing and Midwifery, Mashhad Medical Sciences, Islamic Azad University, Mashhad, Iran; ^7^Islamic Azad University of Medical Sciences, Tehran, Iran; ^8^School of Medicine, Tehran University of Medical Sciences, Tehran, Iran

**Keywords:** irritable bowel syndrome, brain connectivity, IBD, IBS, FA, fractional anisotropy

## Abstract

**Background and aim:**

The neurological processes responsible for irritable bowel syndrome (IBS) pathophysiology and its clinical potentials are not fully understood. The current study aimed to examine white matter microstructural abnormalities and the reasons behind white matter impairment in individuals with irritable bowel syndrome by performing a meta-analysis of diffusion tensor imaging studies.

**Methods:**

PubMed, Scopus and Web of Science were searched until April 2024. Chosen articles based on our defined eligibility criteria were extracted for the data relating to fractional anisotropy and brain connectivity. Webplot digitizer was used to extract digital data. We used the latest version of STATA(ver18) to meta-analyze the data. Quality assessment of studies was done using a critical appraisal tool. Egger’s test for minor study effects assessed the publication bias.

**Results:**

543 IBS cases and 472 healthy controls were included in this study. The mean age of the case and control group was 35.2 ± 17.4 and 33.6 ± 15.8 (mean ± SD), respectively. There was no statistically significant difference in age between groups (*p* > 0.05). Analyzed Standard mean difference using a fixed model for Fractional anisotropy of regions of interest (ROI) associated with sensory processing, such as the thalamus, insula, primary somatosensory cortex, dorsal cingulum and the fornix in selected studies showcased decreased white matter interactivity in case group however this decrease was not statistically different [SMD −88, 95%CI (−1.32, −0.44), *p* > 0.05].

**Conclusion:**

Further investigation is necessary to ascertain whether the modified structural connectivity mentioned in this study is a contributing factor to IBS, an outcome of the condition, a risk factor for it, or, more probably, a consequence of a mutually influential relationship between the changes observed in the white matter tract and IBS symptoms.

## Introduction

IBS, or irritable bowel syndrome, is a prevalent illness that affects the relationship between the brain and the gut. Its prevalence rates range from 1.1 to 45% worldwide, with most Western countries and Asian populations seeing rates between 5 and 10% (Lovell and Ford, 2012). IBS is characterized by persistent stomach pain that occurs regularly and is accompanied by changes in bowel movements without any identifiable physical illness (Simrén et al., 2019). The symptoms of IBS can be incapacitating in a minority of patients but are generally mild to moderate in the majority of affected persons. According to this description, additional commonly linked physical or internal pain and suffering, together with anxiety and depression, are referred to as comorbid illnesses (Drossman, 2016).

IBS has been attributed to altered gastrointestinal motility, visceral hypersensitivity, and psychosocial factors, but recent studies suggest that there is a dysregulation of the brain-gut axis in IBS (Öhman and Simren, 2007). In particular, functional MRI (fMRI) studies (Kwan et al., 2005) and recent studies of cortical gray matter point to dysfunction of emotional and attentional processing of pain in IBS. Furthermore, the subjective nature of pain perception underlines the importance of individual differences such as personality and coping strategy.

Understanding that brain areas do not function independently but as a complex network is essential. The perception of pain results from the integrated activity within this network. The anterior/midcingulate cortex (ACC/MCC), primary and secondary somatosensory cortex (S1, S2), insular cortex (IC), thalamus (Th), and prefrontal cortex (PFC) are the six cortical areas most frequently associated with pain-evoked activity during acute stimulation in humans (Chen et al., 2011).

These identical areas have exhibited distinct responses in patients as opposed to healthy individuals in investigations of persistent pain syndromes, such as migraine, heart pain, fibromyalgia (FM), chronic back pain, temporomandibular disorder, and IBS.

Multiple studies have also documented anomalies in the gray and white matter structure within these specific brain regions in individuals with chronic pain syndromes, including chronic back pain, fibromyalgia, chronic tension-type headache, temporomandibular dysfunction, and irritable bowel syndrome. Consequently, it seems that there are irregularities in the functioning of chronic pain, which are accompanied by abnormalities in the structure of gray and white matter. As a result, developing novel methods to assess the integrity of white matter is stimulating a new area of research in chronic pain of IBS patients. Diffusion tensor imaging (DTI) has gained popularity as a method for evaluating the integrity and connectivity of the brain. One specific value produced from DTI, fractional anisotropy (FA), is frequently used to estimate the microstructural integrity of white matter (Chen et al., 2011).

The presence of persistent symptoms in people with IBS and the lack of effective treatments necessitate continuous attempts to comprehend the causes and perpetuation of symptoms in these individuals. Neuroimaging is a method used to examine the central mechanisms in patients with IBS, which can provide insights into the functioning of the brain-gut axis and its connection to the expression of symptoms. Prior investigations have produced essential discoveries, but ongoing research and technology advancements necessitate a reevaluation of the progress achieved in the sector. However, little is known about white matter abnormalities in patients with IBS, and the current literature does not agree with these changes in the WM tract. Therefore, in this study, we aimed to assess the integrity of white matter in IBS patients through a systematic review and meta-analysis approach.

## Methods

The current study is a systematic review and meta-analysis that adheres to the principles outlined in the PRISMA checklist (Page et al., 2020). The study protocol has been registered within the Open Science Framework (OSF) (DOI 10.17605/OSF.IO/NAJ7Y).

### Search strategy

Two researchers independently searched PubMed, Scopus, and Web of Science for articles published up to April 2024. They used specific search terms including (“irritable bowel* “) OR (IBD) OR (IBS) OR (“Colitis”) AND (“White Matter”) OR (tract) OR (“tract alteration”) OR (“tract change”) OR (“brain connectivity”) OR (“fractional anisotropy”) AND ((((DTI OR (“Diffusion Tensor Imaging”) OR (“Diffusion Tensor Magnetic Resonance Imaging”) OR (Tractography). This search strategy included a mix of Medical Subject Headings (MeSH) and text terms. Additionally, they checked the reference lists of the included articles and relevant reviews and meta-analyses for any additional relevant publications (Table 1).

**Table 1:** Search strategies and the result of the search procedure.

Database	Search strategy	Results
PubMed	(((((((“irritable bowel syndrome “[Title/Abstract]) OR (“Irritable Bowel Syndrome”[Mesh])) OR (IBD[Title/Abstract])) OR (IBS[Title/Abstract])) OR (Colitis[Title/Abstract])) OR (“Colitis”[Mesh])) AND (((((((“White Matter”[Mesh]) OR (“white matter”[Title/Abstract])) OR (tract[Title/Abstract])) OR (“tract alteration”[Title/Abstract])) OR (“tract change”[Title/Abstract])) OR (“brain connectivity”[Title/Abstract])) OR (“fractional anisotropy”[Title/Abstract]))) AND ((((DTI[Title/Abstract]) OR (“Diffusion Tensor Imaging”[Mesh])) OR (“Diffusion Tensor Magnetic Resonance Imaging”[Title/Abstract])) OR (Tractography[Title/Abstract]))	12
WOS	(((TS = (“irritable bowel syndrome “)) OR TS = (IBD)) OR TS = (IBS)) OR TS = (colitis) AND (((((TS = (“White Matter”)) OR TS = (tract)) OR TS = (“tract alteration”)) OR TS = (“tract change”)) OR TS = (“brain connectivity”)) OR TS = (“fractional anisotropy”) AND (((TS = (DTI)) OR TS = (“Diffusion Tensor Imaging”)) OR TS = (“Diffusion Tensor Magnetic Resonance Imaging”)) OR TS = (tractography)	26
Scopus	(TITLE-ABS-KEY(“irritable bowel syndrome “) OR TITLE-ABS-KEY(IBD) OR TITLE-ABS-KEY(IBS) OR TITLE-ABS-KEY(Colitis)) AND (TITLE-ABS-KEY(“White Matter”) OR TITLE-ABS-KEY(tract) OR TITLE-ABS-KEY(“tract alteration”) OR TITLE-ABS-KEY(“tract change”) OR TITLE-ABS-KEY(“brain connectivity”) OR TITLE-ABS-KEY(“fractional anisotropy”)) AND (TITLE-ABS-KEY(DTI) OR TITLE-ABS-KEY(“Diffusion Tensor Imaging”) OR TITLE-ABS-KEY(“Diffusion Tensor Magnetic Resonance Imaging”) OR TITLE-ABS-KEY(Tractography))	24

### The inclusion and exclusion criteria

The selection of eligible articles was based on specific criteria. Inclusion criteria comprised an original, peer-reviewed research paper, a human observational study, the provision of sample size, and fractional anisotropy in IBS cases and healthy controls; it was written in English. Exclusion criteria involved: repeated or duplicated publications; animal studies; disregarding reviews, abstracts, letters, case reports, or conference abstracts lacking original data; studies that did not FA for case and healthy controls; and studies with outcomes related to neuropsychological dysfunction and studies with a sample size having neuropsychological comorbidities were excluded due to confounding DTI results.

### Study selection and data extraction

We used the RAYYAN intelligent tool for systematic reviews to screen the search results (Ouzzani et al., 2016). Titles and abstracts from 7,069 articles obtained from our search strategy were independently and mindlessly screened by two reviewers (MAA, SSRS.). The duplicate records were removed using the same tool. The conflicts were resolved by a third reviewer (FN) using RAYYAN’s compute rating feature.

### Quality assessment of studies

Two authors individually evaluated each candidate article and extracted the relevant information, including the surname of the first author, publication year, country or region, sample size, age and gender distribution of participants, Region of Interest (ROI), fractional anisotropy, BMI, Education years duration of disease, and DTI metrics.

### Risk of bias assessment

The JBI critical appraisal tool evaluated the articles’ methodological quality. Two reviewers independently conducted the quality assessment of all included articles. Any discrepancies were deliberated between the two reviewers, and if a consensus could not be reached, a third reviewer intervened to resolve the disagreement.

### Statistical analysis

STATA ver18 was used to conduct the study analysis. A meta-analysis used Fractional Anisotropy (FA) data as mean ± SD. A random effects model calculated the mean difference and 95% confidence intervals (CIs). A random effects model was also used to combine the study-specific Standardized Mean Difference (SMD) to determine the pooled estimate of the difference in FA of different tracts between IBS cases and control groups. Heterogeneity was assessed using the Chi-square and I-square tests. A subgroup analysis was performed to investigate the factors contributing to heterogeneity. Data points from graphical representations in studies were extracted using WebPlot Digitizer (Automeris LLC, Frisco, Texas). All statistical analyses were two-tailed, with significance at a *p* value <0.05.

### Publication bias assessment

The study examined publication bias using Egger’s regression, and when Egger’s regression identified significant bias (*p* < 0.05), a trim and fill analysis was used to estimate the potential missing effect sizes and to determine a revised overall effect.

## Results

### Study selection and characteristics

**Figure 1:**
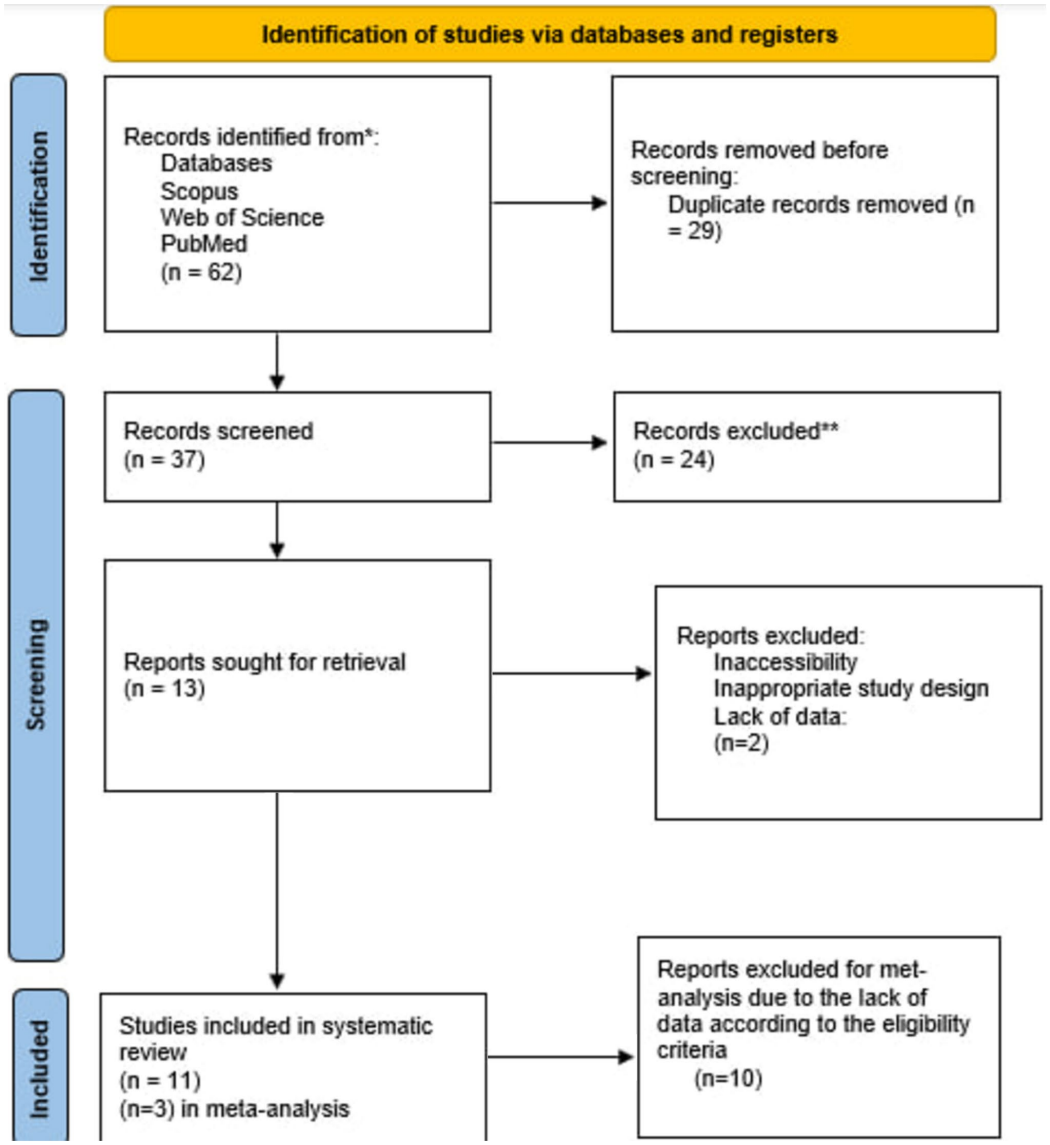
Flow diagram of the study selection procedure.

The curated search strategies yielded a total number of 62 studies across chosen databases (Figure 1). After removing 29 duplicates, the remaining 33 articles were screened by their title and abstracts. Finally, 22 articles were excluded, and 11 studies were incorporated in the systematic synthesis of our study; of these 11 studies, three were assessed analytically appropriate for the meta-analysis.

### Findings

543 IBS cases and 472 healthy controls were included in this systematic review. The mean age of the case and control group was 35.2 ± 17.4 and 33.6 ± 15.8 (mean ± SD), respectively. There was no statistically significant difference in age between groups (*p* > 0.05). The geographical distribution of included studies revealed four studies in China (Fang et al., 2017; Qi et al., 2016; Nan et al., 2018; Zhao et al., 2018), three studies in the USA (Hubbard et al., 2018; Irimia et al., 2015; Ellingson et al., 2013), and the rest were conducted in Canada (Chen et al., 2011), Japan (Chiba et al., 2020), Greece (Zikou et al., 2014), and Sweden (Grinsvall et al., 2021). The summary findings and characteristics of the included studies are demonstrated in Table 2.

**Table 2:** Summary characteristics and findings of the included studies.

	Author	Year	Country	Type of Study	Population (number of cases and number of controls)	Age	Sex	Education (years)	Duration of disease	Region of interest (ROI)	DTI metrics	Findings
1	Grinsvall et al. (2021)	2021	Sweden	Case–control	IBS low somatization (*N* = 31) IBS high somatization (*N* = 35) Total Case (*N* = 66) Control (*N* = 31)	Mean Age IBS low somatization (34.1 ±11.6) IBS high somatization (31.9 ± 8.1) Control (31.5 ±9.4)	IBS low somatization (*n* = 13/18: M/F) IBS high somatization (*n* = 5/30: M/F) Total Case (*n* = 18/48: M/F) Control (*n* = 11/20: M/F)	–	–	Anterior cingulate cortex Midcingulate cortex Amygdala Hippocampus Hypothalamus Anterior insula Middle insula Posterior insula Prefrontal cortex Precentral gyrus Postcentral gyrus Supplementary motor area Thalamus Putamen Cerebellum Midbrain Superior temporal gyrus Inferior parietal lobule	3 Tesla Philips Achieva MR scanner 8-channel head coil T1-weighted 3D TFE gradient echo Magnetization-prepared rapid acquisition gradient echo (MP-RAGE) sequence TR = 7.0 ms TE = 3.2 ms flip angle = 9${{}^{\circ}}^{�}$ Inversion recovery delay of 900 ms shot 2,200 ms The bandwidth of 241 Hx/pixel Voxel size was 1× 1 × 1 mm3, FOV 256 _ 220 _ 176 mm3 Transverse slice orientation None sense TGMV (total gray matter volume)	-The left cerebellum, anterior insula, and prefrontal cortex subregions were more centrally located and connected in those with IBS high somatization
2	Chiba et al. (2020)	2020	Japan	Case–control	Case (*N* = 12) Control (*N* = 7)	Median Age Case: 31–67 (47) Control: 25–79 (31)	Case (*n* = 7/5: M/F) Control (*n* = 6/1: M/F)	–	–	Caudate nucleus Putamen Globus pallidus Thalamus Substantia nigra Red nucleus PAG	3 T scanner Eight-channel head coil Single-shot spin-echo echo-planar imaging (EPI) T1-weighted T2-weighted Fluid-attenuated inversion recovery (FLAIR) Repetition time/echo Time, 4,500/110 ms Motion-probing gradients, 20 directions Values,0, 1,000, and 2,500 s/mm^2^ Field of view, 24 cm Matrix size, 128 × 128 Reconstructed matrix size, 256 × 256 Slice thickness, 3.0 mm without interslice gaps Number of slices, 36 Number of excitations, 4 Reduction factor of parallel imaging, 2 Acquisition time, 12 min, 18 s FA MK MD	-Patients with FGIDs showed diffuse white matter decreased MD - No discernible changes in MK and FA - No differences in deep gray matter Globus pallidus FA values significantly connected significantly negatively with the SF-8 mental component summary.
3	Zhao et al. (2018)	2018	China	Case–control	Case (*N* = 18) Control (*N* = 12)	Mean Age Case: (79.3 ± 5.0) Control: (76.2 ± 7.7)	Case (*n* = 14/4: M/F) Control (*n* = 10/2: M/F)	–	–	Anterior cerebellum lobe Posterior cerebellar lobe Cerebellum nodules Occipital lobe Precentral gyrus Inferior occipital gyrus Limbic lobe Hippocampus Insula Left supramarginal gyrus Middle temporal gyrus Frontal lobe Middle frontal gyrus Angular gyrus hippocampus Insula cingulate gyrus Superior frontal gyrus Medial frontal gyrus Supramarginal gyrus Superior temporal gyrus Callosum Internal capsule Caudex cerebri Fornix Upper corona Superior longitudinal fasciculus External capsule Cingulate gyrus	GE Discovery MR 750 3.0 T 12-channel head coil Sagittal T1WI scan Axial T1WI and T2WI Fluid-attenuated inversion recovery (FLAIR) Diffusion-weighted imaging (DWI) 1-Sagittal T1WI scanning: • TR = 2000 ms • TE = 18 ms • FOV = 240 × 240 mm^2^ • Layer thickness = 5 mm • Matrix size = 320 × 240 2. Axial T1WI: • TR = 2000 ms • TE = 17 ms • FOV = 200 × 230 mm^2^ • Layer thickness = 6 mm • Matrix size = 320 × 168 3. Axial T2WI: • TR = 3,500 ms • TE = 95 ms • FOV = 200 × 230 mm^2^ • Layer thickness = 6 mm • Matrix size = 256 × 256 4. Axial FLAIR: • TR = 9,000 ms • TE = 102 ms • FOV = 200 × 230 mm^2^ • Layer thickness = 6 mm • Matrix size = 256 × 190 5. DWI of axial magnetic resonance: • TR = 27 ms • TE = 20 ms 6. High-resolution whole-brain scan: • TR = 82 ms • TE = 3.2 ms • Flip angle = 12° • Layer thickness = 1.0 mm • FOV = 256 × 256 mm^2^ • Matrix size = 256 × 256 • Voxel size = 1 × 1 × 1 mm^3^	-The insula, superior temporal gyrus, frontal lobe, hippocampus, medial frontal gyrus, superior frontal gyrus, and limbic lobe all had greater GMVs in the IBS group than in the other brain regions. -Both the supramarginal and angular gyrus, The brainstem, fornix, internal capsule, corpus callosum, and upper corona of the IBS group showed reduced fractional anisotropy - IBS patients had higher mean diffusivity in the cingulate gyrus, corpus callosum, upper corona, internal capsule, external capsule, fornix, and superior longitudinal fasciculus.
											7. Diffusion-weighted imaging (DWI): • TR = 8,600 ms • TE = 84 ms • Layer thickness = 1.5 mm • FOV = 212 × 212 mm^2^ • Matrix size = 128 × 128 • Voxel size = 1.65 × 1.65 × 1.5 mm^3^ FA MD	
4	Hubbard et al. (2018)	2018	USA	Case–control	Case (*N* = 16) Control (*N* = 16)	Mean Age Case: (16.29 ±1.78) Control: (16.24 ±1.89)	Case (*n* = 4/12: M/F) Control (*n* = 4/12: M/F)	–	4.125 ± 2.699	Cingulum bundle	MRI Scanner: Siemens 3-T TrioTim Syngo Head Coil: 32-channel Sequence: Simultaneous multi-slice generalized autocalibrating partially parallel acquisition (GRAPPA) echo planar imaging Axial Slices: 70 Field of View (FOV): 220 mm^2^ Matrix Size: 110 × 110 Slice Thickness: 2 mm Resolution: 2 mm^3^ isotropic Repetition Time (TR): 4600 ms Echo Time (TE): 89 ms Diffusion Gradients: 64 directions *b*-value: 1,000 s/mm^2^ GRAPPA Acceleration Factor: *R* = 2 Non-diffusion Weighted Volume (b0): 0 s/mm^2^ FA MD RD	- Observed white matter (WM) abnormalities in adolescents with irritable bowel syndrome (IBS) in the right dorsal cingulum, as indicated by reduced fractional anisotropy (FA). However, Measures of illness severity had no bearing on this decline in WM FA.
5	Nan et al. (2018)	2018	China	Case–control	FC (*n* = 18) IBS-C (*n* = 20) Total Case (38) Control (*N* = 19)	Mean Age FC: (21.11 ± 1.28) IBS-C: (21.9 ± 1.41) Control: (22.74 ± 1.19)	All participants were female	–	FC: (5.1525± 3.0233) IBS-C: (5.5083±2.3067)	Corpus callosum External capsule Corona radiata Superior longitudinal fasciculus	Scanner model: 3-T Signa GE MRI scanner (GE Healthcare, Milwaukee, WI, USA) Number of diffusion sensitizing directions: 30 directions *b*-value for diffusion sensitizing directions: 1,000 s/mm^2^ *b*-value for non-diffusion weighted image: 0 s/mm^2^ Repetition time/echo time: 6,800 ms/93 ms Field of view: 240 mm Matrix: 128 × 128 Number of slices: 45 slices Slice thickness: 3 mm without gap Imaging technique: Single-shot echo planar imaging Repeat acquisition with same settings: Done. FA RD MD AD	-IBS patients showed FA and RD aberrations in the corpus callosum Direct -Comparisons showed only RD differences in the corona radiata and superior longitudinal fasciculus - FA and RD in the corpus callosum were significantly associated with abdominal pain, while FA in CR and SLF were associated with evacuation length and incompleteness.
6	Fang et al. (2017)	2017	China	Case–control	Case (*N* = 21) Control (*N* = 21)	Mean age Case: (41.82 ± 11.92) Control: (35.91 ± 14.76)	Case (*n* = 14/7:M/F) Control (*n* = 11/10: M/F)	Case: (10.29 ± 3.05) Control: (11.48 ± 4.03)	4.92 ± 3.07	Whole brain WM tract Microstructural Decrease AD in: Corpus callosum Internal capsule	MRI scanner: Philips 1.5-T MR scanner T1-weighted 3D sequence parameters: TR (repetition time) = 25 ms TE (echo time) = 4.1 ms Data matrix = 231 × 232 Field of view (FOV) = 230 × 230 Flip angle = 30° Slice thickness = 1 mm Number of axial slices = 132 DTI (Diffusion Tensor Imaging) parameters: Number of diffusion gradient directions = 32 Diffusion gradient strength (b-value) = 800 s/mm^2^ Number of non-collinear directions = 32 TR = 10,793 ms TE = 62 ms FOV = 230 × 230 mm^2^ Matrix size = 128 × 128 Voxel size = 2 × 2 × 2 mm^3^ Slice thickness = 2 mm Slice gap = 0 mm FA RD MD AD	-Individuals with IBS have a reduced (FA) in the splenium of the corpus callosum, the right retrolenticular area of the internal capsule, and the right superior corona radiata. -They also have increased (MD) in the splenium and body of the corpus callosum, the right retrolenticular area of the internal capsule, the right superior corona radiata, and the right posterior limb of the internal capsule. -they have significantly increased (AD) in the splenium of the corpus callosum, the bilateral retrolenticular area of the internal capsule, and the left posterior limb of the internal capsule.
7	Qi et al. (2016)	2016	China	Case–control	Case (*N* = 65) Control (*N* = 67)	Mean Age Case: (34.00 ± 11.82) Control: (31.21± 10.70)	Case (*n* = 49/16:M/F) Control (*n* = 51/16: M/F)	Case: (13.29±4.70) Control: (14.46±3.22)	3.51±4.03	Ventral ACC (anterior cingulate cortex) Inferior parietal lobule Thalamus Inferior occipital/Cerebellumlobes Cuneus Posterior cingulate cortex Lingual gyrus	MRI instrument: 3 Tesla MR **Acquisition of high-resolution structural images**: Sequence: Magnetization-prepared rapid acquisition gradient-echo Orientation: Sagittal Parameters: Repetition time/echo time (TE): 2300 ms/2.98 ms Flip angle: 9 degrees Field of view (FOV): 256 mm × 256 mm Matrix size: 256 × 256 Slice thickness: 1 mm Number of slices: 191 **Acquisition of resting-state fMRI (rs-fMRI) data:** Sequence: Single-shot, gradient-recalled echo-planar imaging Parameters: TR/TE: 2,000 ms/30 ms FOV: 240 mm × 240 mm Flip angle: 90 degrees Matrix: 64 × 64 Voxel size: 3.75 mm × 3.75 mm × 4 mm Number of axial slices: 30, aligned along the anterior–posterior commissure Number of volumes: 250 **Acquisition of diffusion tensor images:** Sequence: Spin echo-based echo-planar imaging Parameters: TR/TE: 4100 ms/93 ms FOV: 240 mm × 240 mm Matrix: 128 × 128 Voxel size: 1.8 mm × 1.8 mm × 4 mm Number of diffusion directions: 20 b-value: 1000 s/mm^2^ Number of volumes: 20 with diffusion weighting, one without diffusion weighting Voxel-mirrored homotopic connectivity (VMHC) FA	-Individuals with IBS exhibit increased interhemispheric functional connectivity between bilateral thalami, cuneus, posterior cingulate cortices, lingual gyri, and inferior occipital/cerebellum lobes. Conversely, their inferior parietal lobules and bilateral ventral anterior cingulate cortices (vACC) are less connected. -Depression and anxiety did not affect the VMHC difference in vACC.
8	Irimia et al. (2015)	2015	USA	Case contol	Case (*N* = 33) Control (*N* = 56)	Mean Age Case: (38.7 ± 10.4) Control: (38.6 ± 11.51)	Case (*n* = 19/14: M/F) Control (*n* = 23/33: M/F)	–	13.68±10.66	Left and right viscerotropic portions of the primary somatosensory cortex (S1) Cortical locations innervated by WM circuitry	Scanner: A Trio Tim scanner For MRI imaging: Repetition time (TR) = 2 s Echo time (TE) = 28 milliseconds Slice thickness: 2 millimeters For DTI imaging: Repetition time (TR) = 9.4 s Echo time (TE) = 88 milliseconds Slice thickness: 2 millimeters Number of gradient directions: 68 Matrix size: 128 × 128 Field of view: 256 millimeters No interslice gap FA	-The primary somatosensory cortex (S1) of HC and IBS participants showed notable variations in both left and right viscerotopic regions. -The predictive variable attributed to these substantial differences was the mean FA of WM bundles innervating S1.
9	Zikou et al. (2014)	2014	Greece	Case–control	Case (*N* = 18) Control (*N* = 20)	Mean Age Case: (45.16 ± 14.71) Control: (45.90 ± 15.92)	Case (*n* = 8/10:M/F)	–	9.03 ± 4.21	Right fusiform Left inferior temporal Left fusiform Right inferior temporal Right precentral The right supplementary motor area Right middle frontal Left superior parietal Corticospinal tract Superior longitudinal fasciculus	MRI unit: 1.5-T Gyroscan Intera Coil type: Quadrature head coil **T1-weighted high-resolution (3D):** Voxel size: 1 × 1 × 1 mm TR: 25 ms TE: 4.6 ms Acquisition matrix: 256 × 228 FOV: 220 mm Imaging time: 5 min 43 s **Single-shot multisection spin-echo echo-planar sequence (DTI):** TR: 9,807 ms TE: 131 ms FOV: 230 mm Matrix: 128 × 128 Section thickness: 3 mm Maximum b value: 700 s/mm^2^ Number of diffusion directions: 16 Scanning time: 5 min 34 s **Sagittal FLAIR sequence:** TR: 6,300 ms TE: 120 ms FOV: 250 mm Matrix: 256 × 256 Bandwidth: 336.7 Hz/pixel Section thickness: 6 mm Intersection gap: 0.6 Scanning time: 2 min 50 s FA MD RD AD	-Reduced gray matter volume was seen in several areas in patients with the disease, including the left superior parietal gyrus, right precentral gyrus, right supplementary motor area, right middle frontal gyrus, and fusiform and inferior temporal gyrus. -Axial diffusivity in the right superior longitudinal fasciculus and corticospinal tract was lower than in the controls.
10	Ellingson et al. (2013)	2013	USA	Case control	Case (*N* = 33) Control (*N* = 93)	Mean Age Case: (33.2 ± 10.8) Control: (30.4 ± 10.4)	Case (*n* = 12/21: M/F) Control (*n* = 21/72: M/F)	–	11.5 ± 1.53	Thalamus Basal ganglia Globus pallidus Putamen Sensory Motor white matter tracts Corpus callosum Coronal radiata White matter	**Acquisition protocol 1** Scanner: Siemens Allegra Field strength: 3 T Echo time (TE): 96 ms Repetition time (TR): 7400 ms Matrix size: 96 × 96 Field of view (FOV): 288 mm Slice thickness (gap): 3 mm (0 mm) Number of directions: 64 Number of b = 0 images: 1 **Acquisition protocol 2** Scanner: Siemens Trio Field strength: 3 T Echo time (TE): 92.6 ms Repetition time (TR): 7000 ms Matrix size: 96 × 96 Field of view (FOV): 288 mm Slice thickness (gap): 3 mm (0 mm) Number of directions: 64 Number of b = 0 images: 1 **Acquisition protocol 3** Scanner: Siemens Trio Field strength: 3 T Echo time (TE): 88 ms Repetition time (TR): 9400 ms Matrix size: 128 ×128 Field of view (FOV): 288 mm Slice thickness (gap): 2 mm (0 mm) Number of directions: 61 Number of b = 0 images: 8 FA MD	-Patients with IBS with chronic, recurrent gut discomfort exhibit long-term microstructural alterations in the brain, especially in areas linked to corticothalamic regulation and sensory information integration. -Patients’ fractional anisotropy (FA) is higher in the corpus callosum and frontal lobe areas and lower in the thalamic, basal ganglia, and sensory/motor association/integration regions. -Patients also have increased MD in the thalamus, internal capsule, and coronal radiata and decreased mean diffusivity inside the globus pallidus. - Patients showed gender disparities in FA and MD, whereas healthy controls did not show these differences.
11	Chen et al. (2011)	2011	Canada	Case–control	Case (*N* = 10) Control (*N* = 16)	Mean Age Case: (32.8 ± 10.4) Control: (29.1 ± 7.9)	All participants were female	–	2–20 years	Mid-anterior cingulum (maC) Medial dorsal nucleus of the thalamus (MD) The ventral posterior lateral nucleus of the thalamus (VPL) Primary somatosensory area (S1) Anterior insula (aIC) External capsule adjacent to anterior and posterior insula (aIC-EC, pIC-EC) Fornix	MRI system: 3 T GE MRI system Head coil: 8-channel phased array head coil Sequence: T1-weighted 3D SPGR sequence Field of view (FOV): 240 mm Slice thickness: 1.5 mm Number of slices: 128 Repetition time (TR): 12 ms Matrix size: 256 × 256 Diffusion-weighted MRI: Sequence: Diffusion-weighted MRI B-value: 1000 Directions: 23 Baselines: 2 FOV: 240 mm Slice thickness: 3 mm Number of slices: 55 TR: 14500 ms Matrix size: 128 × 128 FA	- Increase in (FA) in the external capsule and fornix around the right posterior insula in those with IBS. -The left anterior insula FA was linked to pain unpleasantness, but the bilateral anterior insula and lateral thalamus correlated with the degree of chronic pain. -In the exterior capsule, the length of IBS was linked with FA. -In IBS, there was a negative link between pain catastrophizing and cingulum FA, but in controls, there was a positive correlation between pain catastrophizing and external capsule FA.

Of the 11 included studies, all reported changed FA of ROIs in IBS groups compared to healthy controls except for one study that showed no changed FA.

Chiba et al. (2020) reported that Patients with IBS showed diffuse white matter decreased MD, no discernible changes in MK and FA, and no differences in deep gray matter.

Both the supramarginal and angular gyrus, The brainstem, fornix, internal capsule, corpus callosum, and upper corona of the IBS group showed reduced fractional anisotropy, according to diffusion tensor imaging in the study by Zhao et al. (2018).

Hubbard et al. (2018) found white matter (WM) abnormalities in adolescents with irritable bowel syndrome (IBS) In contrast to a healthy cohort in the right dorsal cingulum, as indicated by reduced fractional anisotropy (FA). IBS patients showed FA and RD aberrations in the corpus callosum in Nan et al.’s study (Nan et al., 2018).

In Qi et al.’s research, individuals with IBS exhibit increased interhemispheric functional connectivity between bilateral thalami, cuneus, posterior cingulate cortices, lingual gyri, and inferior occipital/cerebellum lobes (Qi et al., 2016). According to Ellingson et al.’s findings, Patients’ fractional anisotropy (FA) is higher in the corpus callosum and frontal lobe areas and lower in the thalamic, basal ganglia, and sensory/motor association/integration regions (Ellingson et al., 2013).

According to the study by Chen et al., there was an increase in (FA) in the external capsule and fornix around the right posterior insula in those with IBS. The left anterior insula FA was linked to pain unpleasantness, but the bilateral anterior insula and lateral thalamus correlated with the degree of chronic pain. In the exterior capsule, the length of IBS was linked with FA. In IBS, there was a negative link between pain catastrophizing and cingulum FA, but in controls, there was a positive correlation between pain catastrophizing and external capsule FA (Chen et al., 2011).

### Meta-analysis

**Figure 2:**
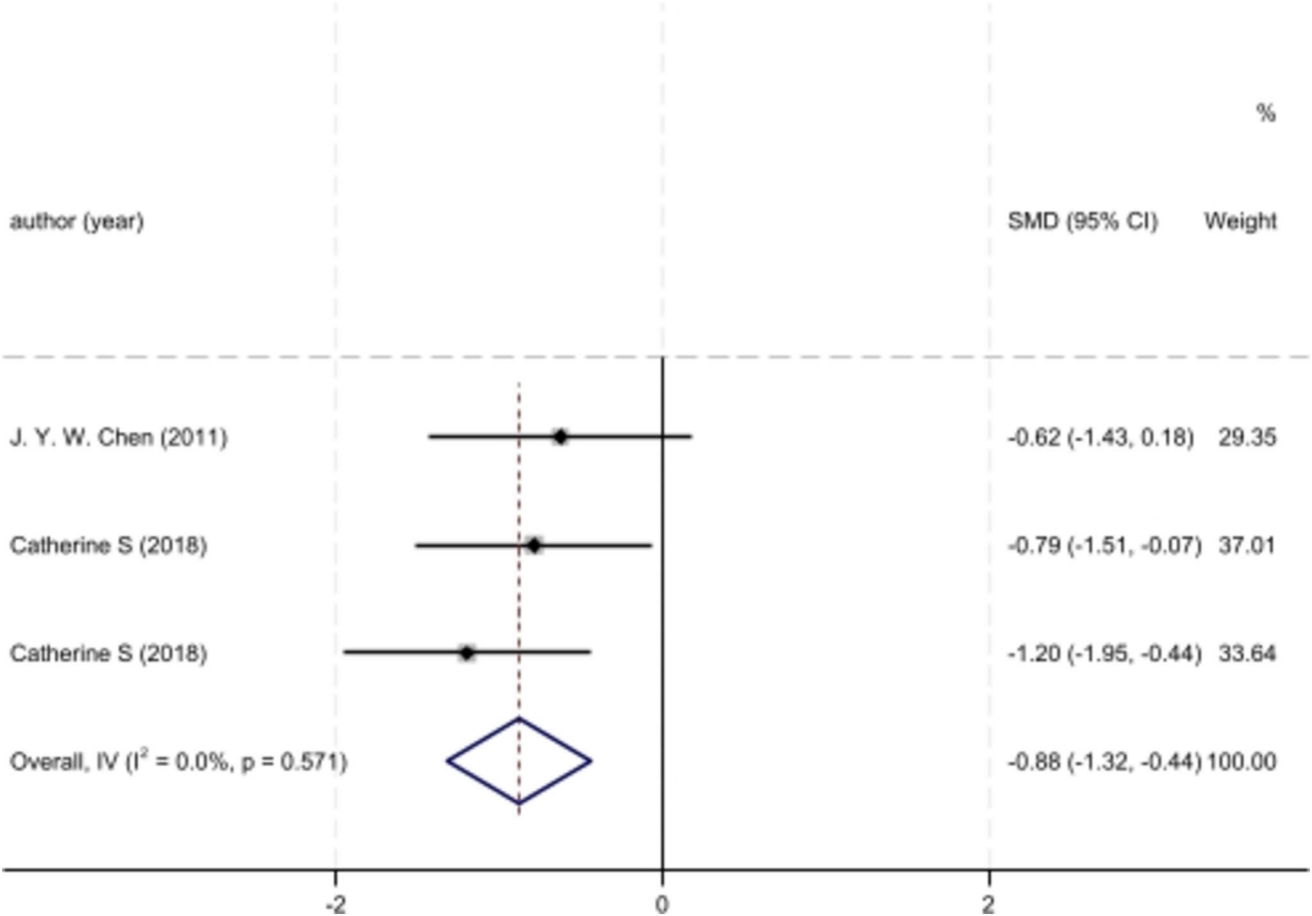
Forest plot of fractional anisotropy alteration analysis across IBS cases and controls of chosen studies.

**Figure 3:**
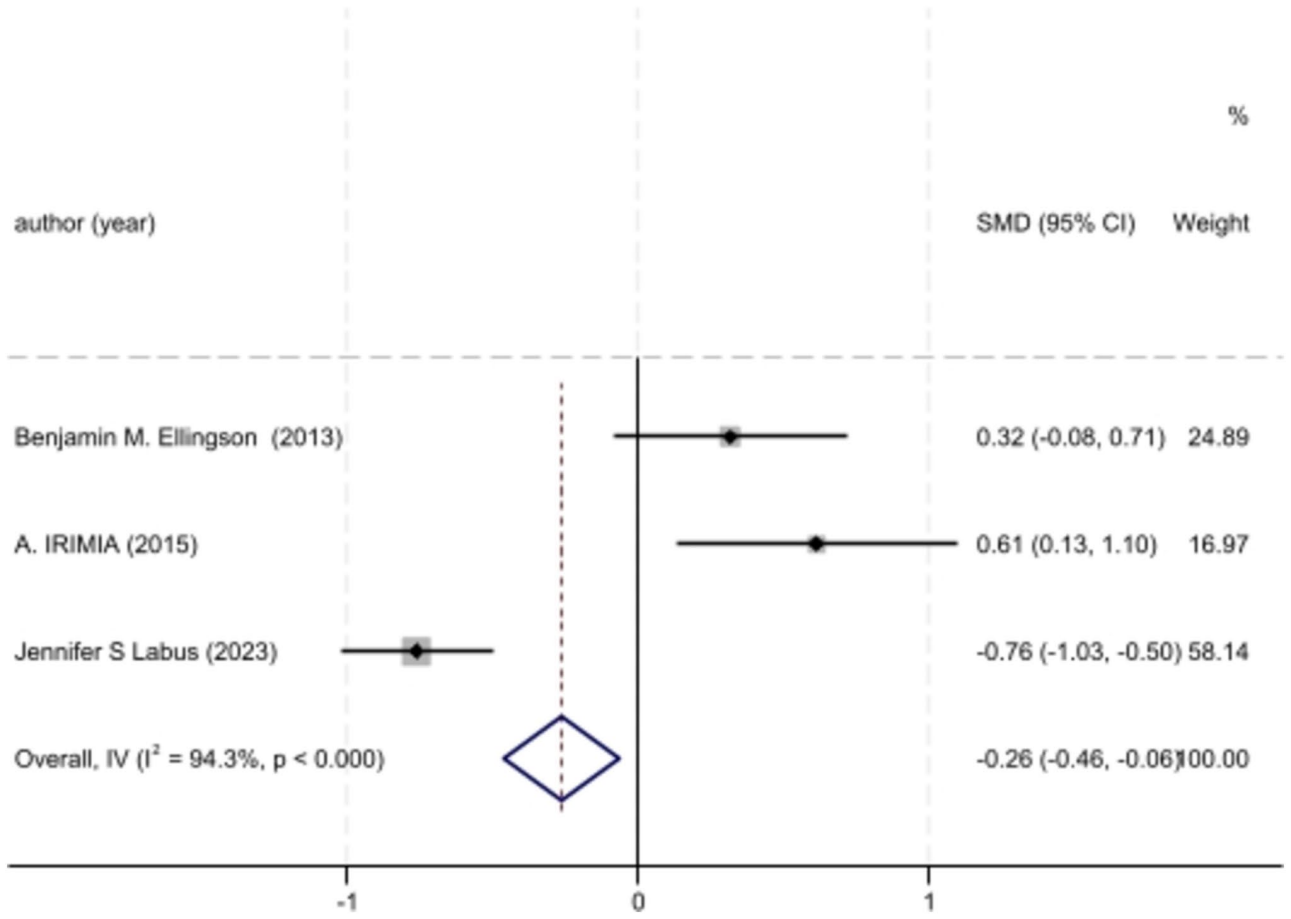
Forest plot of BMI differences across IBS cases and controls of chosen studies.

**Figure 4:**
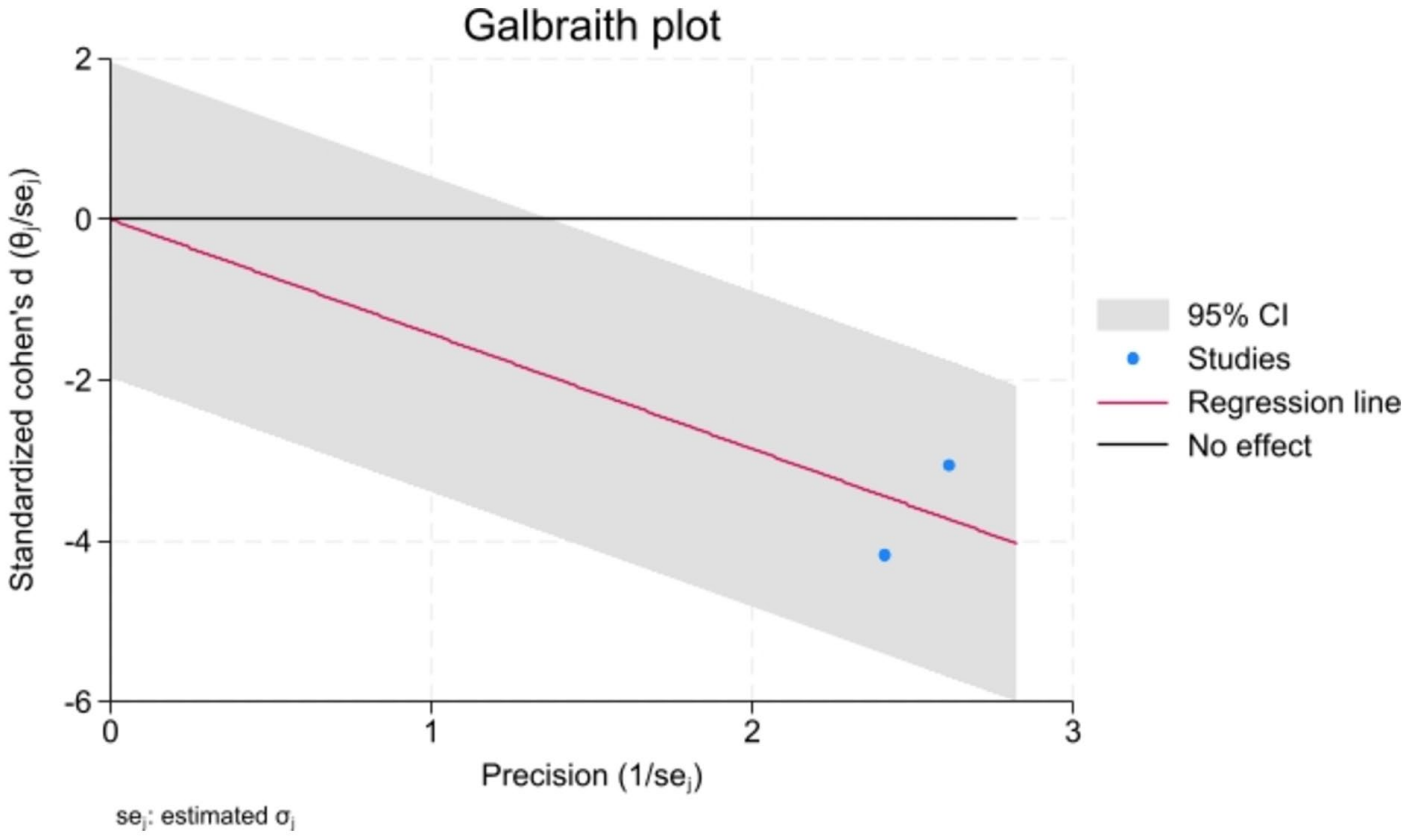
The Galbraith plot for heterogeneity assessment indicated no heterogeneity, further confirmed with the I-squared test (I^2^ < 50%).

**Figure 5:**
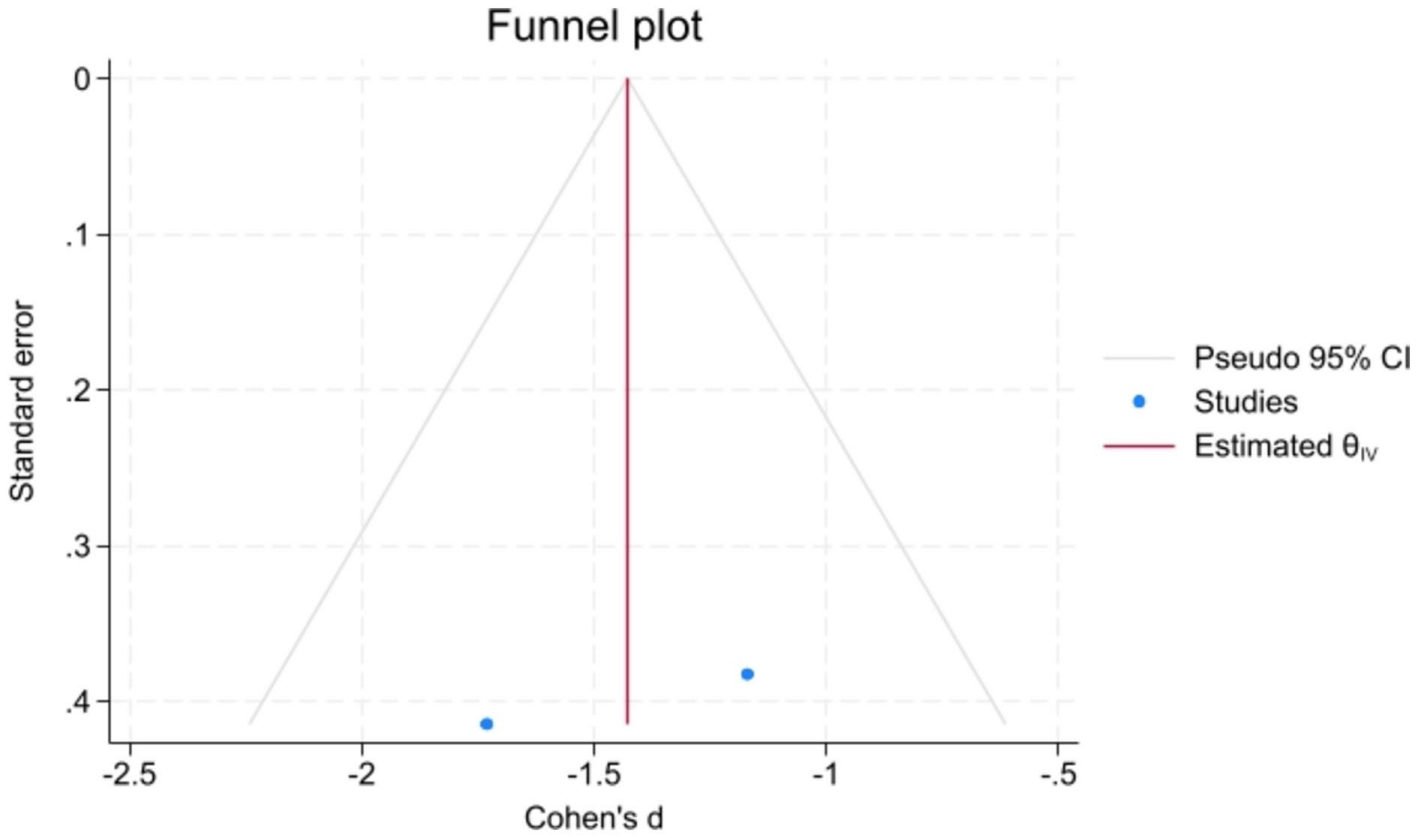
Symmetric demonstration of the funnel plot indicated no publication bias, further confirmed by Egger’s test for publication bias (*p* > 0.05).

Analyzed Standard mean difference using a fixed model for Fractional anisotropy of regions of interest (ROI) associated with sensory processing such as thalamus, insula, primary somatosensory cortex, dorsal cingulum and the fornix in selected studies showcased decreased white matter interactivity in case group however this decrease was not statistically different (SMD −88, 95%CI (−1.32, −0.44), *p* > 0.05) (Figures 2, 3). The I-squared test revealed no significant heterogeneity among studies (I^2^:0.00%) (Figure 4). Results of Egger’s test and funnel plot demonstrated no publication bias (*p* > 0.05, symmetric plot) (Figure 5).

## Discussion

Our research showed that although FA is decreased in IBS patients, it does not differ substantially from the control group’s decline.

Consistent with the findings of this investigation, the great majority of neuropathologies had lower FA with increased MD of WM. However, the FA evaluation is inadequate because it does not account for specific causes of WM integrity degradation, such as changed axon density or diameter and myelination level. To some extent, AD and RD might be indicators of these alterations. AD reflects axons’ width and density and represents axial diffusion’s direction. Decreased AD may indicate damage to specific pathways or axonal degeneration. RD is a measure of myelination degree and indicates the direction of radial diffusion. The RD value is raised by demyelination and hypomyelination and decreased by myelination. Generally speaking, a drop in FA corresponds with either an increase in RD or a decrease in AD. Thus, variations in AD and RD may indicate possible reasons why the integrity of the WM tract may be harmed (Fang et al., 2017; Hubbard et al., 2018). However, we could not expand our analysis to incorporate these divisibility metrics due to a significant lack of data on the MD, RD, and AD of IBS patients.

Similar to numerous other chronic pain diseases and mood disorders, IBS is more prevalent in women, and sex-related disparities in the autonomic, perceptual, and emotional responses of IBS patients to aversive visceral stimuli have been documented. Sex variations in the anomalies in brain function and structure associated with chronic pain are inadequately characterized. The observed sex differences in FA and MD within the IBS cohort indicate more significant white matter alterations in female patients, however these changes are confined to the same locations that differ between healthy controls and IBS patients (Labus et al., 2014). Our study further confirmed these by showing statistically significant difference between white matter integrity alteration between female and male subject (*p* < 0.05).

It is essential to note the technical limits of tractography and DTI, especially concerning the tendency for false positives when employing probabilistic approaches and crossing fibers. To completely understand the differences in tractography between IBS patients and healthy controls, as well as potential confounding factors such psychological distress imposed by the disease itself, future studies are required to use a combination of techniques to alleviate these limitations.

To address these limitations, the implementation and development of a high-efficiency, high-resolution 3D imaging technique for the simultaneous mapping of multiple essential tissue parameters in routine brain imaging, including T1, T2, proton density (PD), ADC, and fractional anisotropy (FA), is crucial. Cao et al.’s suggested DTI-MR fingerprinting method can be used in this regard to advance routine clinical brain imaging from weighted to quantitative imaging, and it is especially beneficial for diffusion investigations, which often have prolonged acquisition times (Cao et al., 2024).

IBS is a complex condition with multiple causes that significantly impact society’s financial and human resources. IBS symptoms might appear at any point along the Brain Gut Axis (BGA) spectrum and have not yet responded to curative medical treatment. Despite significant progress in studying BGA dysfunction in people with IBS, we still do not fully understand how symptoms emerge. Neuroimaging has revealed the physiological distinctions between people with IBS and healthy individuals. Examining variations in neurotransmitter levels, disparities in overall and functional anatomical structure, and the advancing elucidation of a network associated with discomfort caused by rectal distention are crucial scientific advancements in comprehending the pathophysiology of IBS. In order to enhance our comprehension, it will be crucial to utilize appropriate comparator groups, such as individuals with inflammatory bowel disease and psychological illnesses.

The initial results of this study may illustrate the relationship between IBS and brain structure in the examined sample, highlighting that IBS diagnosis in these patients correlates with structural brain differences that may be significant for clinicians. This underscores the potential connection between gastrointestinal diseases, specifically irritable bowel syndrome, and the viscerotropic circuitry of the cerebral cortex.

These studies demonstrate evidence of changes in the brain-gut axis and its potential modulation for therapeutic purposes in patients with IBS.

The importance of this work should be mainly seen as methodological for some reasons. First, altered diffusivity and connection measures will probably need to be addressed in later research with larger samples. A bigger sample size would also aid in performing stronger meta-analyses with higher statistical power in identifying relevant differences between the two groups. Ultimately, more research is required to determine whether the altered structural connectivity described here is a cause of IBS, a result of the condition, a risk factor for it, or, more likely, the result of a reciprocally modulatory relationship between the alterations described in the white matter tract and IBS symptoms.

## Data Availability

The original contributions presented in the study are included in the article/supplementary material, further inquiries can be directed to the corresponding author.
